# Supplementary material for: Chiral phonons in polar LiNbO3
Source: Nat Commun. 2025 Dec 5;17:212. doi: 10.1038/s41467-025-66911-5 (PMC12780136; doi:10.1038/s41467-025-66911-5)
Supplement: Supplementary file 1 — Supplementary Information [file 41467_2025_66911_MOESM1_ESM.pdf]

Supplementary Information for

Chiral phonons in polar $LiNbO_3$

Hiroki Ueda[1,*,†], Abhishek Nag[1,*,†,‡], Carl P. Romao[2,3], Mirian García-Fernández[4], Ke-Jin Zhou[4,X], and Urs Staub[1,*]

[1]*Center for Photon Science, Paul Scherrer Institute, Villigen, Switzerland.*

[2]*Department of Materials, ETH Zurich, Zurich, Switzerland.*

[3]*Department of Materials, Faculty of Nuclear Sciences and Physical Engineering, Czech Technical University in Prague, Czech Republic.*

[4]*Diamond Light Source, Didcot, UK.*

[*]Correspondence authors: hiroki.ueda@psi.ch, abhishek.nag@ph.iitr.ac.in and urs.staub@psi.ch

[†]Equally contributed to this work.

[‡]Present address: Indian Institute of Technology, Roorkee, India.

[X]Present address: University of Science and Technology of China, Anhui, China.

RIXS energy map

Figures S1**a** and S1**d** represent the same X-ray absorption spectrum (XAS) data as Fig. 2**a** but with indicators of the incident photon energies for the resonant inelastic X-ray scattering (RIXS) energy map measurements. The RIXS maps displayed in Figs. S1**b** and S1**e** show phonon resonance at the incident photon energy around 530.85 eV and 535.25 eV, which are sensitive to the hybridization of O 2*p* with the t_{2g} bands and e_g bands of Nb 4*d*, respectively. Interestingly, we observed distinct resonance behavior and polarization contrast between these two photon energies. Figs. S1**c** and S1**f** show high-resolution and high-statistics RIXS scans at $\mathbf{q}_1$ [= (0.1, –0.2, 1)] for the two photon energies. While only the ~100 meV phonon peak is strongly resonant at the lower energy, phonon peaks at three different energy losses discussed in the main text are clearly visible at the higher energy. This may imply that only the phonon mode around 100 meV significantly affects the hybridized orbitals between O 2*p* and the t_{2g} bands of Nb 4*d*. A distinctively large mode effective charge of this high-energy phonon mode, found in Fig. S6, may also play a role in the large RIXS cross-section. No clear circular contrast is found at 530.85 eV (see Fig. S1**c**) at the peak around 100 meV, in contrast to 535.25 eV (see Fig. S1**f**). This might be due to the different symmetries between the two Nb 4*d* bands. The rotation of orbitals due to chiral phonon excitation may only affect a minority of the electric quadrupole moments. This is similar to the fact that even though a

screw-axis forbidden reflection is sensitive to the chirality of a crystal structure, which is visible via circular contrast in resonant diffraction intensities, not all the screw-axis forbidden reflections exhibit circular contrast [1] because a reflection index selects which electric quadrupole moment to contribute to the scattering event.

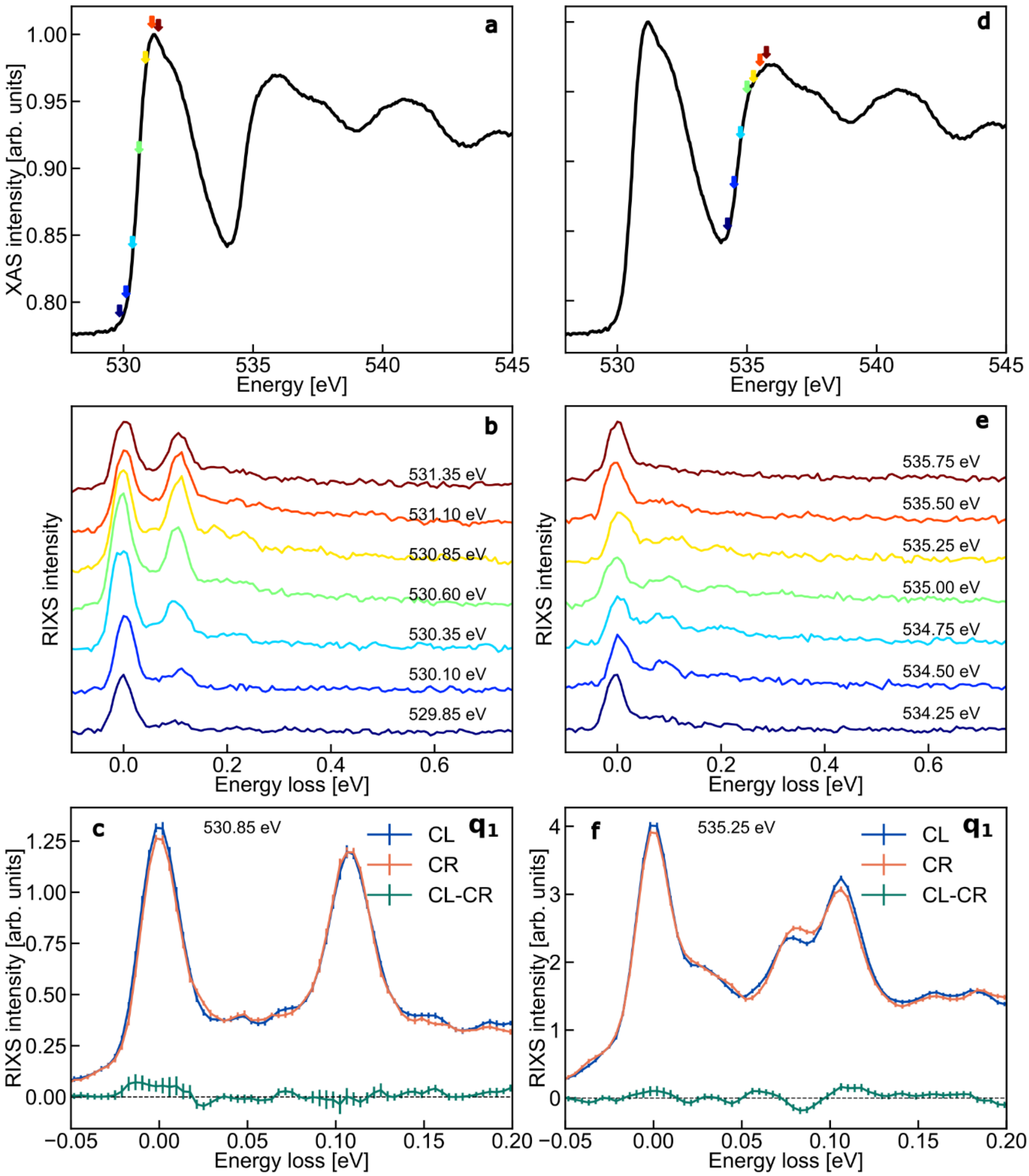

Fig. S1 | **RIXS energy map. a**,**d**, XAS, the same data as the one shown in Fig. 2**a** with indicators of the photon energies used for the RIXS energy map measurements. **b**,**e**, RIXS energy maps at the energies indicated in **a** and **d**, respectively. **c**,**f**, RIXS spectra with

circular polarization at the incident photon energy of 530.85 eV and 535.25 eV, respectively, and the corresponding CD. The error bars in an RIXS spectrum are the standard deviation of individual scans.

Phonon band structure from DFT calculations for $LiNbO_3$

Density-functional theory (DFT) calculation was used to determine the angular momentum and the associated magnetic moments of the phonons arising from the circular motions of the ions (dynamical multiferroicity). These quantities were calculated following the methods described in Ref. [2]. Figure S2 shows arrow plots of the phonon angular momentum texture around the experimentally measured energies. Due to the high density of chiral phonon bands (Fig. S3), in Fig. S2, we have integrated over the phonon bands weighted by a Gaussian centered on the measured energy (as in Figs. 3**d**-3**f** in the main text):

$$\mathbf{J}(\mathbf{q}) = \sum_i \mathbf{J}_i\,(\mathbf{q})\ \exp\{-[E_i(\mathbf{q}) - E_{\text{meas}}]^2/(2\sigma^2)\},\quad \text{(S1)}$$

where $\mathbf{J}_i$ and E_i are the phonon angular momentum vectors and energies of the phonon bands at a given $\mathbf{q}$ vector. The standard deviation accounting for the instrument energy resolution (~23 meV full width at half-maximum) is described as σ. The reversal of phonon chirality between $\mathbf{q}_1$ and $\mathbf{q}_3$ due to c glide symmetry, which coincides with the plane spanned by the hexagonal reciprocal lattice vectors $\mathbf{a}^*$ and $\mathbf{c}^*$ or $\mathbf{b}^*$ and $\mathbf{c}^*$, is clearly visible in Figs. 3**d**-3**f**. Along these planes, $\mathbf{J}$ is only nonzero in directions perpendicular to the plane. Consequently, since the character of the phonons varies smoothly through reciprocal space, the phonon chirality $\mathbf{J}\cdot\mathbf{q}$ is diminished at points close to glide planes, including the area near the center of the plots (along $\mathbf{c}^*$).

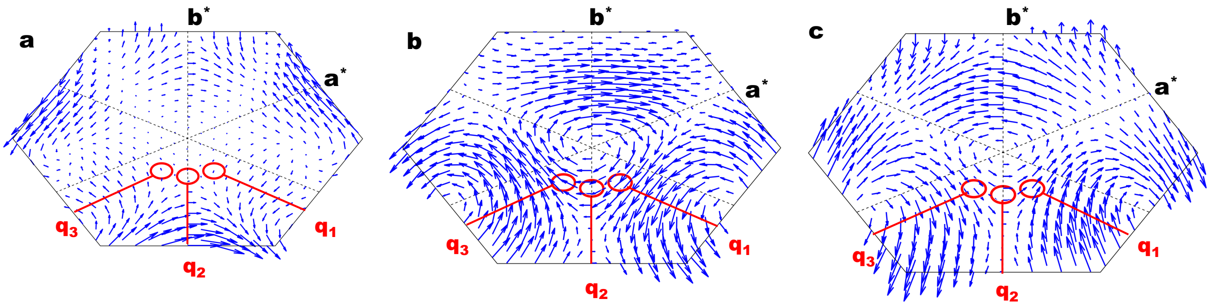

Fig. S2 | **Arrow plots of phonon angular momentum. a-c**, Arrow plots of phonon angular momentum ($\mathbf{J}$) centered at **a**, E_1 (25 meV), **b**, E_2 (77 meV), and **c**, E_3 (106 meV), shown for the surface corresponding to (h k 1) in hexagonal reduced coordinates in reciprocal space. To account for the instrumental resolution, the plots consider contributions from each

phonon weighted by a Gaussian centered at E_1, E_2, or E_3, with full width at half-maximum of 23 meV. The size of the arrows is normalized to the maximum magnitude of **J** in each plot (**a**: 0.13 $\hbar$, **b**: 0.24 $\hbar$, **c**: 0.08 $\hbar$).

Figures S3 and S4 show the phonon angular momentum and magnetic moment, respectively, of all bands at special points and paths throughout the Brillouin zone, in addition to the **q** points measured in the RIXS experiment. Figure S5 is a diagram of the rhombohedral Brillouin zone, with the special points Γ (0 0 0), T (−0.5 −0.5 −0.5), H (0.5 −0.235 0.235), L (0.5 0 0), S (0.368 −0.368 0), and F (0.5 0 0.5) marked, as well as the reciprocal lattice vectors of the hexagonal lattice as a guide for conversion between the two settings. As Fig. S3 shows, the phonons in $LiNbO_3$ generally have some degree of chirality, although none of them are highly chiral. The magnitude of the circular polarization only reaches approximately 0.5, compared to close to 1 in α-quartz [2]. The large number of bands close in energy means there are few regions of momentum–energy parameter space with large contrast between left and right circularly polarized phonons, as consistent with the experimental results shown in Fig. 2. The phonon magnetic moments (Fig. S4) are small (*ca.* 0.1 μ_n) and similar to those in α-quartz [2]. The larger gyromagnetic ratios of the ions in $LiNbO_3$ compared to α-quartz are compensated for by the smaller magnitude of the phonon circular polarization.

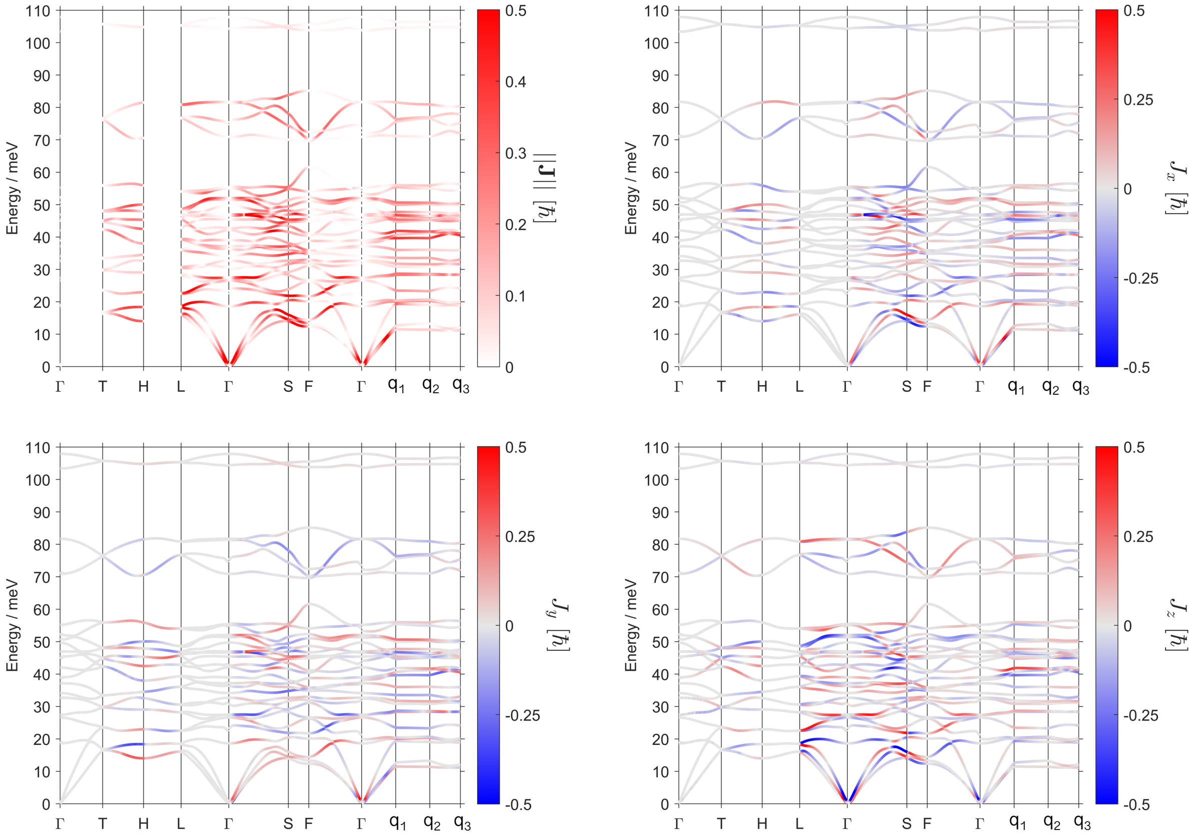

Fig. S3 | **Phonon band structure with angular momenta.** The phonon band structure of $LiNbO_3$, with bands coloured by the magnitude of their phonon angular momentum (**J**) (top left), and its Cartesian components J_x, J_z, and J_y (clockwise from top right). The Cartesian directions are defined such that the hexagonal lattice vector a is aligned with x and c with z. Special points in and paths through the Brillouin zone were chosen following Ref. [3].

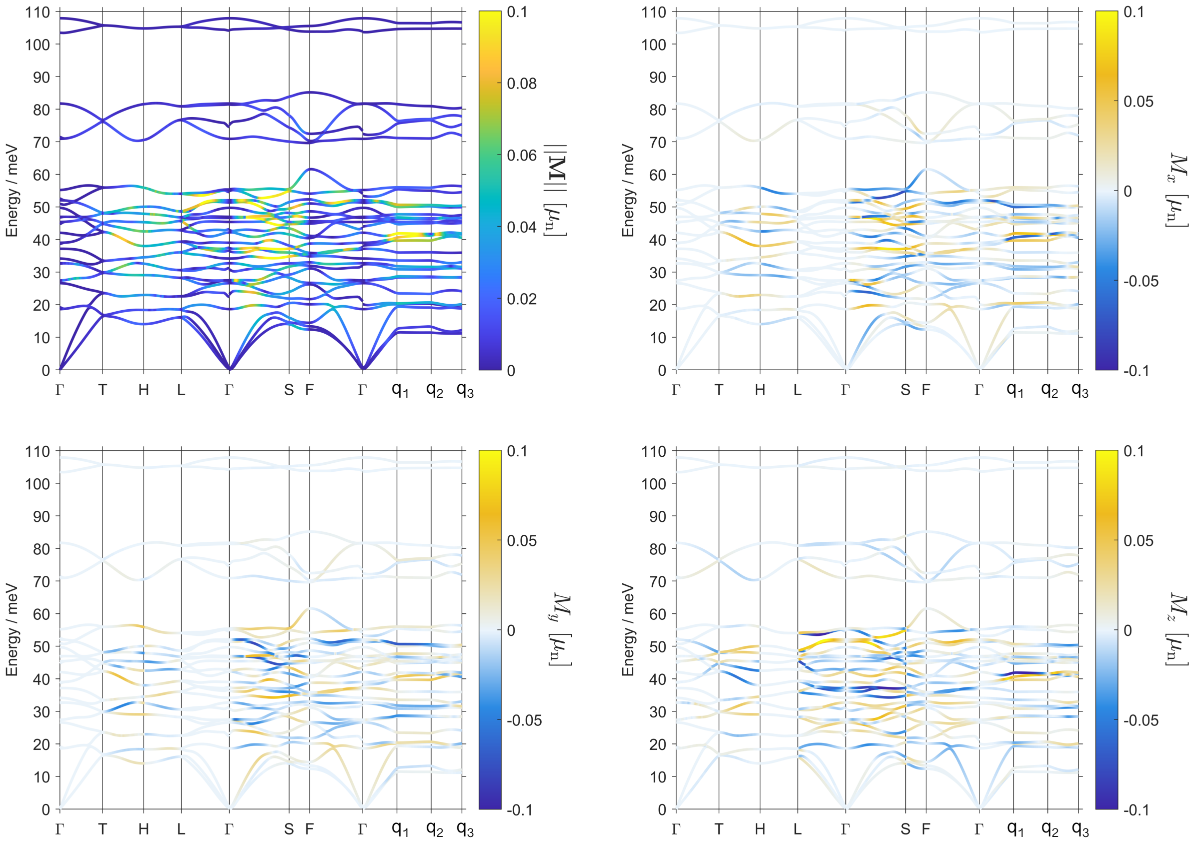

Fig. S4 | **Phonon band structure with magnetic moments.** The phonon band structure of $LiNbO_3$, with bands coloured by the magnitude of their phonon magnetic moment (**M**) (top left), and its Cartesian components M_x, M_z, and M_y (clockwise from top right), in units of the nuclear magneton (μ_n). The Cartesian directions are defined such that the hexagonal lattice vector a is aligned with x and c with z. Special points in and paths through the Brillouin zone were chosen following Ref. [3].

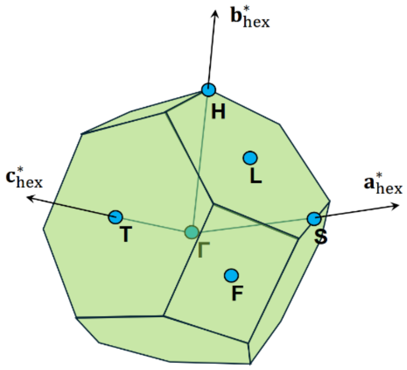

Fig. S5 | **Rhombohedral Brillouin zone.** A diagram of the first Brillouin zone of $LiNbO_3$, corresponding to the rhombohedral primitive cell. Special points and the directions of the hexagonal reciprocal lattice vectors are marked. Adapted from Ref. [3].

The mode effective charge, which measures the magnitude of the electronic distribution perturbed by the phonon eigendisplacement [4] and is therefore related to the strength of its interaction with light, is shown in Fig. S6. Unlike in the case of α-quartz [2], in $LiNbO_3$ there are low levels of contrast in the mode effective charges for the modes with relatively large degree of phonon chirality at the measured **q** points, precluding the identification of specific phonons contributing to the RIXS CD signal from analysis of the mode effective charge.

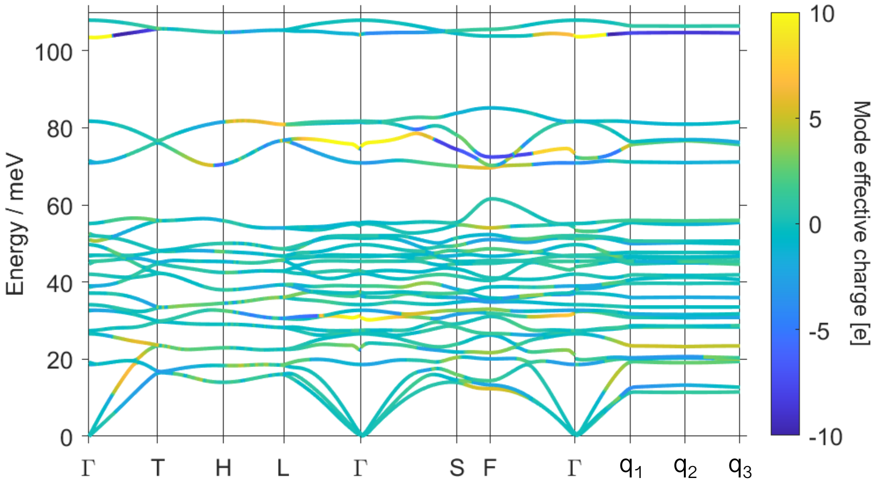

Fig. S6 | **Phonon band structure with mode effective charge.** The phonon band structure of $LiNbO_3$, with bands coloured by the magnitude of their mode effective charges, in units of the elementary charge (*e*). Special points in and paths through the Brillouin zone were chosen following Ref. [3].

The birefringence effect in RIXS circular dichroism

The birefringence, well-known in optics, describes splitting a single ray into two rays, called an ordinary ray and an extraordinary ray. The splitting occurs for different refractive indices between the two orthogonal directions perpendicular to the optical path. The ordinary ray and extraordinary ray directions deviate most for a maximal difference in the refractive index. Thus, they are not along the vertical or horizontal polarization directions in an experimental setup in general. The birefringence is absent in isotropic media, such as cubic materials, or when the incident ray is along the principal axis of uniaxial crystals, i.e., hexagonal, tetragonal, and trigonal materials. This is the reason why Raman spectroscopy

with circular polarization is limited for chiral phonons with a small momentum transfer along the principal axis [5]. The splitting is less significant in the X-ray range because the X-ray refractive index in materials is very close to 1, in contrast to the optical range. Even though the splitting is still tiny at an atomic resonance, the resonance enhances the anisotropy large enough to affect an X-ray polarization state, i.e., the relative phase between two split X-rays, because of the short wavelength of X-rays. Circular polarization is described by two oscillating orthogonal electric fields with equal amplitude and a phase shift of π/2. Once the birefringence affects their relative phase, the polarization state is no longer circular but becomes elliptical. Note that the ellipsoid axes generally differ between the nominal two incident circular polarization states, C+ and C−, because the ordinary ray and extraordinary ray directions do not match the vertical or horizontal polarization direction in an experimental setup. Therefore, nominal circular polarization C+ and C− have a different amplitude ratio of vertical and horizontal polarization before the X-ray scattering happens inside the material. The X-ray scattering amplitude usually depends on linear X-ray polarization, even for isotropic charge scattering. As a result, X-ray scattering intensities depend on incident circular polarization states as if the scattering were circular dichroic [6,7].

The phonon peak at E_3 shows strong linear dichroism compared to the other phonon peaks, as found in Fig. S7, consistent with the eigendisplacements being almost pure linear and having a large mode effective charge (see Supplementary Video 3 and Fig. S6). This indicates that the birefringence effect can be significant at the high-energy phonon peak.

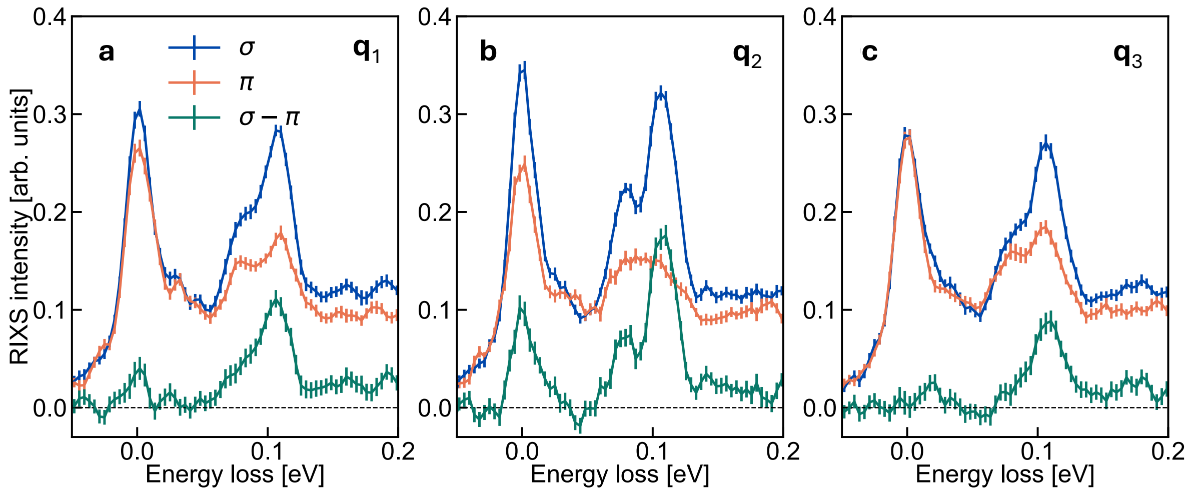

Fig. S7 | **Linear dichroism of the phonon peaks in $LiNbO_3$.** RIXS spectra taken with σ and π polarized incident X-rays at **a**, $\mathbf{q_1}$, **b**, $\mathbf{q_2}$ and **c**, $\mathbf{q_3}$. The error bars in an RIXS spectrum are the standard deviation of individual scans.

Here, we investigate the symmetry of the birefringence signals. Since the electric field of light is oscillating, the birefringence effect needs to be twofold rotationally symmetric along [001], in addition to the threefold rotational symmetry from the crystal structure. The combination of these rotational symmetries is sixfold. The RIXS spectra at $\mathbf{q}_1$, $\mathbf{q}_2$, and $\mathbf{q}_3$, shown in the main text, have been collected at the incidence angle of ~45°. Therefore, the birefringence effect can contribute to the CD signals. Since the sixfold rotational symmetry connects $\mathbf{q}_1$ and $\mathbf{q}_3$, the CD signals due to the birefringence should be identical between the two momentum points. The CD signals due to chiral phonons respect the crystal symmetry, which results in threefold rotational symmetry and flipped circular contrast between the two momentum points. Hence, our experimental data, displayed in Figs. 2**b** and 2**d**, which show the reversed circular contrast between $\mathbf{q}_1$ and $\mathbf{q}_3$ at E_1 and E_2, indicate that the dominant origin of circular contrast originates from the chirality of the phonons. Besides, fitting by two sinusoidal functions following either threefold or sixfold rotational symmetry, as done in Fig. 3**i**, allows us to distinguish the chiral phonon signal from the birefringence effect.

Insensitivity to cycloidal (two-dimensional chiral) phonons

Figure S8 shows the experimental geometry when performing the RIXS measurements at $\mathbf{q}_2$. The *c* glide plane is parallel to the scattering plane, and the phonon angular momentum associated with a two-dimensional chiral or cycloidal phonon ($\mathbf{J}_{\text{phon}}$) is perpendicular to the plane. A circularly polarized X-ray photon brings angular momentum ($\mathbf{J}_{\text{phot}}$) parallel to the scattering plane. Hence, the angular momentum of the incident photon is orthogonal to the phonon angular momentum of the cycloidal phonon. This geometry does not allow angular momentum transfer between the photon and phonon, and no CD signals are expected in the RIXS intensities. This consideration also applies to the other momentum points where we have collected RIXS data. As a result, RIXS CD in our experimental geometry is solely sensitive to chiral phonons.

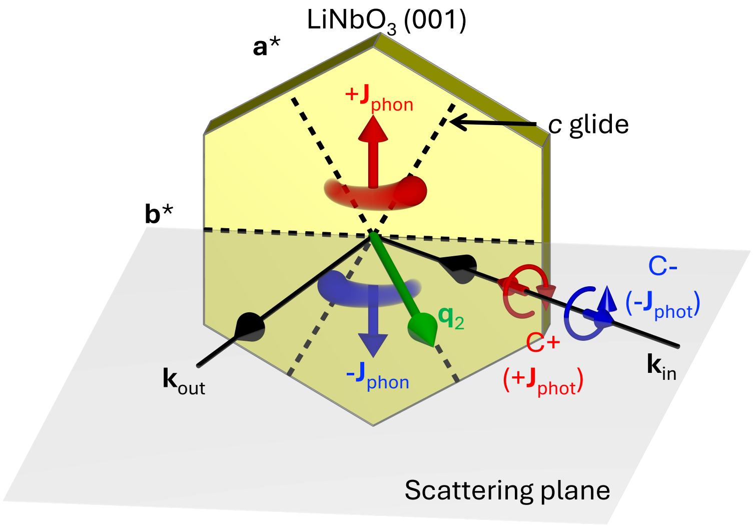

Fig. S8 | **Experimental geometry.** The incident X-ray polarization propagates perpendicular to the phonon angular momentum induced by a cycloidal phonon at $\mathbf{q}_2$.

Fitting polar plots

We fit the polar plots shown in Figs. 3g-3i using two sinusoidal functions: one with threefold symmetry (amplitude: a_3) representing the chiral phonon contribution, and one with sixfold symmetry (amplitude: a_6) representing the birefringence effect. Table S1 summarizes the fit parameters.

Table S1 | Fit amplitudes for the polar plots of RIXS-CD signals shown in Figs. 3g-3i (E_1 – E_3).

	E_1	E_2	E_3
a_3	$(1.9\pm0.6)\times10^{-2}$	$(2.0\pm0.7)\times10^{-2}$	$(-2.0\pm0.7)\times10^{-2}$
a_6	$(-6\pm5)\times10^{-3}$	$(-5\pm6)\times10^{-3}$	$(-2\pm0.5)\times10^{-2}$

RIXS spectra at $\mathbf{q}_4$ and $\mathbf{q}_5$

RIXS spectra collected at $\mathbf{q}_4$ and $\mathbf{q}_5$, which were used to generate the data shown in Fig. 3, are shown in Fig. S9.

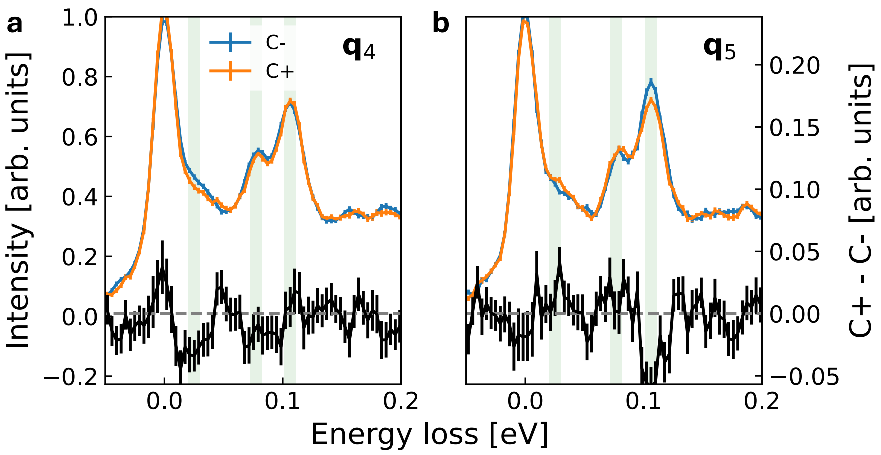

Fig. S9 | **RIXS spectra at different momentum points.** RIXS spectra taken with circular polarizations at **a**, $\mathbf{q}_4$ and **b**, $\mathbf{q}_5$. The error bars in an RIXS spectrum are the standard deviation of individual scans.

Reference

1. Ueda, H., Skoropata, E., Burian, M., Ukleev, V., Perren, G. S., Leroy, L., Zaccaro, J., and Staub, U., Conical spin order with chiral quadrupole helix in $CsCuCl_3$. *Phys. Rev. B* **105**, 144408 (2022). DOI: https://doi.org/10.1103/PhysRevB.105.144408
2. Ueda, H., García-Fernández, M., Agrestini, S., Romao, C. P., van den Brink, J., Spaldin, N. A., Zhou, K.-J., Staub, U., Chiral phonons in quartz probed by X-rays. *Nature* **618**, 946-950 (2023). DOI: https://doi.org/10.1038/s41586-023-06016-5
3. Hinuma, Y., Pizzi, G., Kumagai, Y., Oba, F., and Tanaka, I., Band structure diagram paths based on crystallography. *Comp. Mat. Sci.* **128**, 140 (2017). DOI: 10.1016/j.commatsci.2016.10.015
4. Gonze, X., and Lee, C., Dynamical matrices, Born effective charges, dielectric permittivity tensors, and interatomic force constants from density-functional perturbation theory. *Phys. Rev. B* **55**, 10355 (1997). DOI: https://doi.org/10.1103/PhysRevB.55.10355
5. Ishito K., Mao, H., Kousaka, Y., Togawa, Y., Iwasaki, S., Zhang, T., Murakami, S., Kishine, J., and Satoh, T., Truly chiral phonons in α-HgS. *Nat. Phys.* **19**, 35-39 (2023). DOI: https://doi.org/10.1038/s41567-022-01790-x
6. Joly, Y., Collins, S. P., Grenier, S., Tolentino, H. C. N., and De Santis, M., Birefringence and polarization rotation in resonant x-ray diffraction. *Phys. Rev. B* **86**, 220101(R) (2012). DOI: https://doi.org/10.1103/PhysRevB.86.220101
7. Nag, A., Perren, G. S., Ueda, H., Boothroyd, A. T., Prabhakaran, D., García-Fernández, M., Agrestini, S., Zhou, K.-J., and Staub, U., Circular dichroism in resonant inelastic x-ray scattering from birefringence in CuO. *Phys. Rev. Res.* **7**, L022047 (2025). DOI: https://doi.org/10.1103/PhysRevResearch.7.L022047
