# Supplementary material for: Chiral phonons in polar LiNbO3
Source: Nat Commun. 2025 Dec 5;17:212. doi: 10.1038/s41467-025-66911-5 (PMC12780136; doi:10.1038/s41467-025-66911-5)
Supplement: Supplementary file 2 — Description of Additional Supplementary Files [file 41467_2025_66911_MOESM2_ESM.pdf]

Description of Additional Supplementary Files:

Title: Supplementary Video 1

Description: [001]-view of the phonon eigenmode at E_1 and $\mathbf{q}_1$.

Title: Supplementary Video 2

Description: [001]-view of the phonon eigenmode at E_2 and $\mathbf{q}_1$.

Title: Supplementary Video 3

Description: [001]-view of the phonon eigenmode at E_3 and $\mathbf{q}_1$.
